# Evaluation of a brief online multi-index assessment for predicting increased psychotic-like experiences in the community: A perceptual, cognitive and affective approach.

**DOI:** 10.1016/j.scog.2025.100357

**Published:** 2025-03-12

**Authors:** Caroline Cullen, Keith Gaynor, Klaus Kessler

**Affiliations:** a School of Psychology, University College Dublin, Belfield, Dublin 4, Ireland; b DETECT, Early Intervention Psychosis Service, Blackrock, Co. Dublin, Ireland

**Keywords:** Psychotic-like experiences, Cognition, Online assessment, Emotion dysregulation, Community sample

## Abstract

Research has shown that impairments in perception, reasoning, and social cognition are evident across the psychosis continuum and are implicated in the transition from subclinical symptoms to clinical psychosis. In this pilot feasibility study, a brief computerised assessment of visual perception, reasoning, social cognition and emotion dysregulation was administered to 157 adults in the community alongside self-report measures of psychotic-like experiences. The feasibility, reliability, and the predictive validity of the assessment tool were examined. The assessment procedure was feasible, evidenced through high completion rates. However, reliability estimates were suboptimal for online assessment measures. Self-reported visual perception and state emotion dysregulation predicted psychotic-like experiences explaining 53% of the variance when controlling for age. This study provides preliminary evidence that state difficulties with emotion regulation and self-reported visual perception abnormalities can predict increased psychotic-like experiences in the community. Future adaptations could address technological issues encountered with assessment tasks and ensure measures are psychometrically robust when administered online. Brief online assessments hold potential for research of both cognition and affect along the psychosis continuum although caution must be exercised with the chosen methodology.

## 1 Introduction

### 1.1 A continuum model of psychosis

Psychotic-like experiences (PLEs) are subclinical symptoms of psychosis experienced by individuals with no-need-for-care and reflect the core principles of the concept of a psychosis continuum (Johns and van Os, 2001). PLEs are typically transitory and around 80 % of people who experience them do so for only a brief period. Approximately 20 % of people with PLEs experience these persistently, and 7 % of those will develop a psychotic disorder with an annual transition rate of 0.5–1 % (van Os and Reininghaus, 2016). One such model of this trajectory is the psychosis proneness-persistence-impairment model (van Os et al., 2008) whereby many people in the community may have a proneness to brief psychotic experiences, a smaller group has long-term persistent psychotic experiences without distress, and the smallest group are impaired by their experiences and meet criteria for a psychotic disorder. By identifying factors which differentiate people with PLEs and psychotic disorders, research can shed light on the mechanisms that drive the transition from proneness to persistence and persistence to impairment (van Os and Reininghaus, 2016). A key, and very challenging goal of research is to identify what mechanisms may distinguish individuals who will be distressed and impaired by their experiences and those who will not (van Os et al., 2008). Seiler et al. (Seiler et al., 2020) identified that PLEs occur across two axes (i) severity of psychotic experiences and (ii) distress/need-for-care. Understanding the factors which determine severity and distress is fundamental to understanding protective and risk factors. However, despite much research in this area few vulnerability factors have been found to differentiate the PLE and psychotic groups (Peters et al., 2016). Easy-to-administer assessments could identify individuals at risk of psychosis progression, enabling early intervention for more favourable outcomes.

### 1.2 Cognitive subsystems

Cognition is one such area which may determine need for care. A wide range of deficits in perception, reasoning, and affective subsystems are evident in clinical samples (Mollon and Reichenberg, 2017), and impaired cognition may be implicated in the transition from psychosis-proneness to clinical psychosis (Montemagni et al., 2020). There are a range of cognitive subsystems related to psychosis and three are examined within this study alongside emotion dysregulation and were chosen based on prior psychosis research indicating impairments in these areas (Staines et al., 2022). The selected subsystems prioritized evaluation of a diverse range of cognitive abilities while balancing the practical need for efficient and easily self-administered assessments.

#### 1.2.1 Visual perception

Visual perception is a cognitive subsystem involved in information processing fundamental for understanding and adapting to the environment (Qiong, 2017). Discrepancies in bottom-up and top-down visual processing can cause inaccurate internal representations of the external environment and may explain hallucinations (Hemsley, 2005). Visual perception abnormalities can be measured using illusions and self-reports. Adolescents with PLEs have shown increased susceptibility to illusions (Sperandio et al., 2023) while other studies across the psychosis spectrum find mixed results (Ferri et al., 2018; Gupta et al., 2016; Lányi et al., 2024). Self-reported visual perceptual abnormalities are related to earlier onset of illness, greater symptoms, and worse premorbid functioning in clinical patients (Keane et al., 2018).

#### 1.2.2 Jumping to conclusions bias

Jumping to conclusions (JTC) bias is a cognitive reasoning style relating to the propensity to prematurely conclude information about an event in the absence of sufficient information. JTC bias is a suggested cognitive trait marker of psychosis (Díaz-Cutraro et al., 2022) and a contributing factor to the onset and maintenance of delusional ideation (Moritz et al., 2017). Preliminary evidence suggests JTC biases may contribute to the progression and persistence of psychosis (Rauschenberg et al., 2020) while other evidence proposes JTC bias is the result, or consequence of general cognitive impairment rather than involved in the progression of psychosis (Tripoli et al., 2020).

#### 1.2.3 Theory of Mind

Theory of Mind (ToM) is an element of social cognition defined as the ability to attribute mental states to the self and others (Premack and Woodruff, 1978). ToM deficits hold clinical relevance due to their contribution to social functioning impairments and are considered a potential marker of psychosis risk (Bora, 2017). Delusions experienced in psychosis can manifest as maladaptive thoughts that others have negative intentions towards them and may be explained by ToM deficits. In contrast to clinical patients, individuals with PLEs may have suspicions that others mean harm yet realise this is not tied to reality (Zhang et al., 2016).

#### 1.2.4 Emotion dysregulation

The final subsystem examined in this study is the affective component, emotion dysregulation. Several studies suggest that emotion dysregulation may play a role in both the development and maintenance of psychotic disorders (Hardy, 2017; van Rossum et al., 2011; Jannini et al., 2025). Emotion dysregulation is prevalent in clinical populations however less is known in subclinical communities and it is still unclear whether it predicts vulnerability for psychosis (Aldao et al., 2010). A key parallel between clinical and non-help-seeking individuals is less reported distress relating to PLEs and distress is a predictive factor of the transition from subclinical symptoms to psychotic illness (Chapman et al., 2019). The ability to manage emotional responses may be related to management of PLE related distress.

### 1.3 Assessment context

Cognitive assessments require highly trained administrators, can be lengthy and difficult to disseminate to the community in preventative strategies (Vita et al., 2022). Therefore, there is a services need for short, usable digital cognitive assessment tools for screening or for repeated testing over time. Online assessments can address the practical limitations of face-to-face and facilitate research in extensive populations. Equivalent levels of compliance and performance have been found when comparing online vs laboratory-based experiments suggesting experimental research can be conducted remotely with sufficient fidelity (Buso et al., 2021).

### 1.4 Research aims

The aim of this study is to administer a brief online multi-index assessment of cognitive and affective subsystems implicated in psychosis to no-need-for-care individuals with PLEs to be completed through smartphones, PCs, or laptops. The first goal is to examine the reliability and feasibility of this assessment procedure. Secondly, we hypothesize that there will be group differences in task performance between low and high PLE groups, and that performance on cognitive tasks will predict PLE scores. A third and final objective of this study is to examine the relationship between the different aspects of cognition (visual perceptual, jumping to conclusions, theory of mind, emotion dysregulation) to PLEs.

## 2 Materials and methods

### 2.1 Design

This study used a cross-sectional, quantitative online methodology at a single time point. It was a pilot feasibility study of online multi-index assessment. A correlational design examined the predictive validity of cognitive tasks in predicting PLEs. Following refinement, future studies will examine similar tools in clinical samples.

### 2.2 Power analysis

Sample size calculations were performed using G*Power (Faul et al., 2007). Calculations were computed to achieve a small effect size (*f* = 0.15) with 95 % power and resulted in sample sizes of 119 based on logistic regression analysis with 3 predictor variables.

### 2.3 Participants

We recruited adults from the general population with a range of PLEs in line with the recruitment methodology of large multi-national studies (Siddi et al., 2024). The study used two recruitment strategies (i) convenience and (ii) purposive sampling methods. (i) The study was posted on SONA, an online student research participation system at University College Dublin where participants received research credit for taking part. (ii) Participants were also recruited using an advertisement posted in online communities discussing anomalous experiences with moderator permission on platforms such as Reddit and Facebook. Participants were informed the study focused on unusual experiences, emotions, perception & reasoning. Inclusion criteria were age > 18 with sufficient command of English to offer informed consent. Individuals reporting current or lifetime diagnosis of a psychotic disorder were excluded from analysis. Participation in this study was entirely voluntary, and individuals did not receive any financial or other forms of reimbursement for their involvement.

### 2.4 Procedure

Participants viewed detailed information about the study before offering informed consent. Brief demographic questions were followed by questionnaire measures of PLEs, emotion dysregulation, self-report visual perceptual abnormalities, and experimental tasks examining ToM, visual perceptual abnormalities and JTC bias. The study took on average 26-min and data were collected through Microsoft Forms from March–June 2024.

### 2.5 Questionnaire measures

#### 2.5.1 Psychotic-like experiences: the Community Assessment of Psychic Experiences (CAPE-42)

The CAPE-42 (Stefanis et al., 2002) is a reliable and valid tool to examine PLEs in general populations (Mark and Toulopoulou, 2015). For a detailed description of scoring please see https://cape42.homestead.com. For the present study, frequency and distress items were summed to establish an overall score. High and Low PLE Groups were divided above or below the 75th percentile on the Frequency subscale.

#### 2.5.2 Emotion dysregulation: State Difficulties in Emotion Regulation Scale (S-DERS)

This 21-item scale conceptualises emotion dysregulation as a fluid construct rather than a dispositional tendency and proposes emotion dysregulation can vary over time particularly in response to external experiences (Lavender et al., 2016). Total scores were calculated by summing all items after reverse coding and higher scores were indicative of greater state emotion dysregulation.

#### 2.5.3 Self- reported visual perception abnormalities: Bonn Scale for the Assessment of Basic Symptoms

The vision scale is a 17-item subscale of the Bonn Scale interview (Gross et al., 1987) examining lifetime history of visual perceptual abnormalities. The subscale has shown utility in clarifying diagnosis and predicting outcomes in clinical samples (Keane et al., 2018). Total scores were summed, and higher scores indicated greater lifetime visual perceptual abnormalities.

### 2.6 Experimental tasks

#### 2.6.1 Reasoning: Jumping to Conclusions subscale of the Cognitive Biases Questionnaire for psychosis

This task included 6 short vignettes with scenarios reflecting two themes of major relevance to psychosis: anomalous perceptions and threatening events (Peters et al., 2013). Participants were requested to imagine themselves in each situation and forced choice responses were scored 1–3 ranging from absence of bias (1), possible presence of bias (2) and likely presence of bias (3). Scores ranged from 6 to 18, higher scores indicated greater JTC reasoning style.

#### 2.6.2 Perception: Müller-Lyer illusion; Ebbinghaus illusion; Ponzo illusion

Participants were presented with images of the well-known Müller-Lyer, Ebbinghaus and Ponzo illusions (Müller-Lyer, 1889; Titchener, 1905; Ponzo, 1928) Participants scored 1 if they were susceptible to the illusion and 0 if they were not. Scores were summed to a total.

##### 2.6.2.1 Müller-Lyer illusion

Participants were presented with an image where the perceived length of a line depends on the arrowheads on both its sides. Participants indicated if they thought lines were the same length, or if one was longer, indicating susceptibility.

##### 2.6.2.2 Ebbinghaus illusion

Participants viewed two images of orange inner circles surrounded by black outer circles and rated if one circle was bigger than the other, or if they were the same size Participants rating one circle as bigger/smaller were susceptible to the illusion.

##### 2.6.2.3 Ponzo illusion

Participants viewed an image of two identical yellow lines across a pair of converging lines and rated if one was longer, or if they were the same length and if they rated one line longer than the other, they were susceptible to the illusion.

#### 2.6.3 Social cognition: hinting task

This shortened 5-item version (Gil et al., 2012) is derived from the original task (Corcoran et al., 1995) and includes 5 stories involving 2 characters. In each story one character drops a hint and an open question enquired what the character really meant by the hint. Items were scored from 0 to 2. Responses were scored according to SCOPE criteria (Klein et al., 2020) and summed to a total ranging from 0 to 10. Higher scores indicated greater ToM ability. Scoring was completed by two independent reviewers and discrepancies were settled by a third independent reviewer.

### 2.7 Data analysis

Analysis was completed using the Statistical Package for Social Sciences (SPSS) version 29. Missing data <5 % was managed using pairwise/listwise deletion and missing data >5 % was handled with mean/median substitution methods. Assumptions of normality, linearity, multicollinearity, and homoscedasticity were evaluated prior to analysis. Non-parametric Kruskal-Wallis H tests were used for age group differences and Pearson's correlations examined associations between variables. Hierarchical multiple regression was used to assess predictive validity of measures, two-tailed *t*-tests were run to examine group differences.

### 2.8 Ethics

The Human Research Ethics Committee at University College Dublin granted ethical approval. Informed consent was obtained, and all procedures were in accordance with the Helsinki Declaration (World Medical Association, 1996). Although we did not anticipate participation would cause distress, a distress protocol was in place where a range of supports were provided at debrief stages.

## 3 Results

### 3.1 Missing data

One hundred and fifty-seven participants responded. However, ten reported a diagnosis of a psychotic disorder and were subsequently excluded from analysis to ensure a subclinical sample (*N* = 147, *M* age = 45.25). Data were missing at item level and < 5 % for most variables therefore pairwise/listwise deletion was used. Self-report visual perceptual abnormalities and employment status had missing data over 5 % (6.3 %). For visual perceptual abnormalities the mean of observed values was used to replace missing entries and the median for observed values was used to replace missing responses for employment.

### 3.2 Tests of normality

Prior to analysis, normality of distribution was checked with Q-Q plots and examination of skewness. Total scores for the experimental measures and self-report visual perceptual abnormalities were significantly skewed (e.g., skewness greater than ±1). Log_10_ transformation procedures were applied to correct skewness and once transformed, tests of normality were non-significant, and skewness was within +1/−1 parameters.

### 3.3 Descriptive statistics

Participants were predominantly female (*n* = 116, 80.3 %), 17 % were male (*n* = 25) and 0.7 % non-binary (*n* = 1). Participants were more likely to be married (49.3 %), employed full-time (54.2 %) and educated to undergraduate degree level (31.9 %). 30.6 % were Irish, 22.9 % were American, 17.4 % were from the UK while 6.3 % were Australian. 5.6 % were from Europe, 2.1 % were African. Canadian and Asian participants each made up 1.4 % of the sample.

### 3.4 Demographic differences

There were no significant differences between males (*M* = 70.52, *SD* = 16.03) and females (*M* = 71.39, *SD* = 15.26) on overall CAPE-42 scores; *t* (139) -0.26, *p* = .399. Age had a significant negative relationship with CAPE-42 scores (*r* = −0.32, *p* < .001) and there were significant differences in CAPE-42 scores across age groups (*x*^2^ (3) = 16.30, *p* < .001) with the 18–29 group scoring highest (*Mdn* = 73.00), and over 65 s scoring lowest (*Mdn* = 61.00).

### 3.5 Feasibility and reliability of the online assessment measure

Feasibility was evidenced by the recruitment of a large sample for a cognitive assessment (*N* = 137), with an exceptionally high level of measure completion, and very low missing data (<5 %). High completion rates demonstrated ease of use while allowing for appropriate statistics and a wide range of scoring indicated meaningful variation of PLEs in the community. As seen in Table 1, reliability coefficients were strong for all questionnaire measures. However, Cronbach's alpha was borderline minimum acceptable levels for Susceptibility to illusion measures and were significantly below acceptable levels for both ToM and JTC bias tasks.

**Table 1:** Descriptive statistics of key variables.

	Mean	Std. deviation	Range	Median	Skewness	Kurtosis	Reliability
CAPE-42 Frequency	71.10	15.24	86.00	67.00	0.80	0.60	*a* = 0.92
CAPE-42 Distress	36.26	20.83	143.00	33.00	−0.15	0.41	*a* = 0.94
Emotion dysregulation	38.55	12.71	64.00	35.00	0.68	0.12	*a* = 0.91
VPA	4.31	3.74	16.00	3.50	0.93	0.30	*a* = 0.88
Susceptibility to Illusion	1.27	1.15	3.00	1.00	0.27	−1.39	*a* = 0.69
JTC	10.08	1.99	10.00	10.00	0.78	0.57	*a* = 0.51
ToM	8.30	1.37	5.00	10.00	−0.60	−0.39	*a* = 0.34
Age	45.25	15.00	67	46.00	0.10	−0.47	

VPA = visual perception abnormalities, JTC = jumping to conclusions, ToM = Theory of Mind. Skewness and kurtosis figures reported are results following transformation procedures; mean, SD, range, median, are reported from raw data.

### 3.6 High vs Low PLE Groups

As seen in Table 2, JTC showed significant differences between the high/low PLEs group while other cognitive assessments showed none. However, due to low reliability, only cautious conclusions can be drawn. There were significant group differences in questionnaire measures of visual perceptual abnormalities and emotion dysregulation.

**Table 2:** Differences in scores on measures between high and low PLE groups.

	High PLE mean (SD)	Low PLE mean (SD)	T-test (df)	*p* value
*Cognitive assessment measures*				
JTC	11.12 (2.56)	9.76 (1.66)	−2.91 (142)	0.003
ToM	8.26 (1.28)	8.31	0.19 (142)	0.426
Susceptibility to illusion	1.17 (1.11)	1.31 (1.61)	0.56 (142)	0.289

*Questionnaire measures*				
VPA	6.71 (3.90)	3.56 (3.19)	−4.30 (142)	<0.001
Emotion dysregulation	49.27 (17.36)	35.24 (8.60)	−4.69 (142)	<0.001

Note PLE: psychotic-like experiences, JTC: jumping to conclusions, ToM: Theory of Mind, VPA: visual perception abnormalities, ED: emotion dysregulation.

### 3.7 Predictive validity of experimental measures

Correlational analysis was completed on the primary variables. As seen in Table 3, the cognitive assessment had low predictive validity on CAPE-42 scores. ToM and overall susceptibility to illusion scores were not correlated with any other measures. Results showed large significant relationships between CAPE-42 scores, emotion dysregulation total and subscales, self-report visual perceptual abnormalities and JTC.

**Table 3:** Correlations of questionnaire and experimental variables.

	JTC	ToM	Müller-Lyer	Ebbinghaus	Ponzo	Illusion total	VPA	ED
CAPE-42 Frequency total	0.40⁎⁎	−0.06	0.01	−0.09	0.11	0.01	0.56⁎⁎	0.51⁎⁎
CAPE-42 Distress total	0.44⁎⁎	−0.01	0.02	−0.04	0.17	0.06	0.45⁎⁎	0.53⁎⁎
JTC	–							
ToM	−0.06							
Müller-Lyer	0.04	−0.13						
Ebbinghaus	0.02	−0.12	0.45⁎⁎					
Ponzo	0.28⁎⁎	−0.06	0.31⁎⁎	0.50⁎⁎				
Illusion total	0.14	−0.14	0.74⁎⁎	0.84⁎⁎	0.77⁎⁎			
VPA	0.16	−0.17	0.05	0.07	0.13	0.11		
ED	0.43⁎⁎	−0.14	0.03	−0.00	0.09	0.05	0.13	
Age	−0.47⁎⁎	0.10	0.04	0.22⁎⁎	−0.14	0.05	−0.10	−0.43⁎⁎

JTC: jumping to conclusions, ToM: Theory of Mind, VPAs: visual perception abnormalities, ED: emotion dysregulation.

⁎⁎ Correlation is significant at the 0.01 level (2-tailed).

Based on significant correlations, a hierarchical multiple regression assessed the ability of a model made of emotion dysregulation, self-report visual perceptual abnormalities and JTC bias to predict PLE frequency scores after controlling for the influence of age and a second model for PLE distress scores. The overall model explained 53 % of the variance of PLE frequency and 47.5 % of the variance of PLE distress and model summary results are seen in Table 4. For PLE frequency, the final model was statistically significant *F* (4, 137) =38.66, *p* < .001. JTC was not a significant contributor to the final model, when age was included as a control but significantly contributed to the model when age was not controlled for b = 0.152, *p* = .020. For PLE distress, the final model was also statistically significant *F* (4, 137) =30.98, *p* < .001.

**Table 4:** Model summaries of hierarchical linear regressions examining CAPE-42 Frequency and Distress.

	R^2^	*b*	df1	df2	F change	p
*CAPE-42 Frequency*						
Age	0.104	−0.32	1	140	16.21	<0.001
Emotion dysregulation	0.276	0.46	1	139	33.02	<0.001
Self-report VP	0.518	0.50	1	138	69.42	<0.001
JTC	0.530	0.13	1	137	3.52	0.063

*CAPE-42 Distress*						
Age	0.148	−0.39.	1	140	24.33	<0.001
Emotion dysregulation	0.311	45	1	139	32.81	<0.001
Self-report VP	0.455	0.39	1	138	36.67	<0.001
JTC	0.475	0.17	1	137	5.08	<0.001

Model 1 = Age as control, PV = emotion dysregulation, self-report visual perception abnormalities and JTC. DV = CAPE Frequency total.

Model 2 = Age as control, PV = emotion dysregulation, self-report visual perception abnormalities and JTC. DV = CAPE Distress total.

## 4 Discussion

This study administered a brief online multi-index assessment of cognitive and affective subsystems implicated in psychosis in a PLE sample. The feasibility and reliability of the assessment was partially successful as the assessment engaged a large appropriate sample with high completion rates and meaningful data were collected. Low reliability of experimental measures indicated tasks were unreliable measures of the underlying cognitive constructs in the current format. Item level exploratory analysis did not find greater reliability. Attempts to establish a brief assessment compromised validity and several methodological issues were encountered. Considering the suboptimal reliability, testing group differences of the cognitive measures was completed on an exploratory basis. Significant differences were found on the ToM, visual perception abnormalities and emotion dysregulation measures between high and low PLE groups. Self-reported visual perceptual abnormalities and state emotion dysregulation predicted approximately 50 % of PLEs and related distress in this community sample.

### 4.1 Implications & future direction

These findings underscore the necessity for further inquiry into how individuals with PLEs visually perceive the world and how dysregulated affective states impact them. Few studies have examined affective states alongside PLEs (Jannini et al., 2025) and this study is the first to our knowledge to administer S-DERS to subclinical individuals with PLEs and adds to the scant literature of emotion dysregulation along the psychosis continuum. Findings also strengthen rationale for emotion dysregulation as a treatment target in clinical populations. Dialectical Behaviour therapy (DBT) is a well-established treatment that has previously demonstrated efficacy as an intervention for psychosis (Lawlor et al., 2022). Keane (Keane et al., 2018) found visual perceptual abnormalities predicted schizophrenia and the current study offers preliminary evidence that visual perceptual abnormalities predict PLEs in a no-need for care group. Basic symptoms have received significantly less attention compared to positive or negative symptoms but have strong evidence bases and warrant further investigation (Schultze-Lutter et al., 2016). One of the challenges of utilising basic symptoms is a cumbersome interviewer-based assessment (Schultze-Lutter et al., 2012) and the low reliability of historical self-report measures such as the Frankenfurt Complaint Scale (Michel et al., 2016). However, recent research has found interviewer assessment of the visual component of basic symptoms specifically to be reliable and predictive of psychotic symptoms (Diamond et al., 2022) and the current study extends this to self-report visual perceptual abnormalities. If this finding is replicated in larger samples, it offers a potential practical basic symptom screen. Recognising true prodromal symptoms as opposed to broader PLEs is crucial, as the length of time psychosis remains untreated significantly affects the disease's progression and can lead to poorer outcomes (Westhoff et al., 2022).

### 4.2 Strengths & limitations

This study featured cross-cultural representation, diverse age groups and findings present a new line of evidence along the psychosis continuum. Several limitations should be noted. This study lacked a comparison clinical group, and females were overrepresented. Findings should be replicated before assumptions on their generalizability can be made. The current study was also limited by a cross-sectional design and longitudinal designs could track potential progression to clinical status and how this relates to cognition and emotion dysregulation. It was not possible to include a measure of mood in the test battery which presents a further limitation considering the relevance of emotion dysregulation.

Finally, this study did not control for visual acuity, and it was not possible to establish if self-reports of visual perceptual abnormalities were impacted by medical conditions, drug, or alcohol use. Future adaptations could include further objective measures of visual perceptual abnormalities to control for confounding influences.

## 5 Conclusion

A brief online assessment of PLEs in the community was feasible although tasks showed unacceptable reliability in the current format. State emotion dysregulation and self-report visual perceptual abnormalities predicted PLEs and related distress in a non-help-seeking community sample. Brief online assessments show promise although caution must be taken with the chosen methodology.

## CRediT authorship contribution statement

**Caroline Cullen:** Writing – original draft, Methodology, Investigation, Formal analysis, Data curation, Conceptualization. **Keith Gaynor:** Writing – review & editing, Supervision, Methodology, Conceptualization. **Klaus Kessler:** Supervision, Software, Methodology, Conceptualization.

## Declaration of competing interest

The authors declare that they have no known competing financial interests or personal relationships that could have appeared to influence the work reported in this paper.
